# Study on the transcriptome for breast muscle of chickens and the function of key gene *RAC2* on fibroblasts proliferation

**DOI:** 10.1186/s12864-021-07453-0

**Published:** 2021-03-06

**Authors:** Genxi Zhang, Pengfei Wu, Kaizhi Zhou, Mingliang He, Xinchao Zhang, Cong Qiu, Tingting Li, Tao Zhang, Kaizhou Xie, Guojun Dai, Jinyu Wang

**Affiliations:** 1 College of Animal Science and Technology, Yangzhou University, Yangzhou, 225009 China; 2 Joint International Research Laboratory of Agriculture & Agri-Product Safety, Yangzhou University, Yangzhou, 225009 China; 3 Jiangsu Jinghai Poultry Group Co. Ltd., Nantong, 226100 China

**Keywords:** Jinghai yellow chicken, Growth and development, RNA-seq, qPCR, RNAi

## Abstract

**Background:**

Growth performance is significant in broiler production. In the growth process of broilers, gene expression varies at different growth stages. However, limited research has been conducted on the molecular mechanisms of muscle growth and development in yellow-feathered male chickens.

**Results:**

In the study, we used RNA-seq to study the transcriptome of the breast muscle of male Jinghai yellow chickens at 4 (M4F), 8 (M8F) and 12 weeks (M12F) of age. The results showed that 4608 differentially expressed genes (DEGs) were obtained by comparison in pairs of the three groups with Fold Change (FC) ≥ 2 and False Discovery Rate (FDR) ≤ 0.05, and 83, 3445 and 3903 DEGs were obtained separately from M4FvsM8F, M4FvsM12F and M8FvsM12F. Six genes were found as co-differentially expressed in the three age groups, namely *SNCG, MYH1A, ARHGDIB, ENSGALG00000031598, ENSGALG00000035660* and *ENSGALG00000030559*. The GO analysis showed that 0, 304 and 408 biological process (BP) were significantly enriched in M4FvsM8F, M4FvsM12F and M8FvsM12F groups, respectively. KEGG pathway enrichment showed that 1, 2, 4 and 4 pathways were significantly enriched in M4FvsM8F, M4FvsM12F, M8FvsM12F and all DEGs, respectively. They were steroid biosynthesis, carbon metabolism, focal adhesion, cytokine-cytokine receptor interaction, biosynthesis of amino acids and salmonella infection. We constructed short hairpin RNA (shRNA) to interfere the differentially expressed gene *RAC2* in DF-1 cells and detected mRNA and protein expression of the downstream genes *PAK1* and *MAPK8*. Results of qPCR showed that *RAC2*, *PAK1* and *MAPK8* mRNA expression significantly decreased in the sh*RAC2*–2 group compared with the negative control (NC) group. Western Blot (WB) results showed that the proteins of *RAC2*, *PAK1* and *MAPK8* also decreased in the sh*RAC2*–2 group. Cell Counting Kit-8 (CCK-8) and 5-Ethynyl-2′-deoxyuridine (EdU) assay both showed that the proliferation of DF-1 cells was significantly inhibited after transfection of sh*RAC2*–2.

**Conclusions:**

The results of RNA-seq revealed genes, BP terms and KEGG pathways related to growth and development of male Jinghai yellow chickens, and they would have important guiding significance to our production practice. Further research suggested that *RAC2* might regulate cell proliferation by regulating *PAKs/MAPK8* pathway and affect growth of chickens.

**Supplementary Information:**

The online version contains supplementary material available at 10.1186/s12864-021-07453-0.

## Background

Chicken meat has the nutritional characteristics of high protein, low fat and low calorie. Yellow-feathered broilers, as germplasm resources of local breeds in China, are more and more popular with consumers because of their strong disease resistance and delicious meat [1–5]. However, the growth rate of indigenous chicken is slower than that of commercial large white-feathered broilers [5, 6]. In order to improve their growth performance, it is necessary to study the growth and development mechanism regulation. In the breeding of broilers, the ratio of male to female chicken in natural mating is generally 1:8–12, but the ratio in artificial insemination can reach 1:20–30, even up to 1:50. Half of the offspring’s genome comes from the roosters, so their performance has a greater impact on the population growth performance than that of hens. However, limited research has been conducted on the molecular mechanisms of muscle growth and development in yellow-feathered male chickens.

With the advent of post-genomic era, genomics technologies such as transcriptome, proteome, metabonome have emerged, among which RNA-seq was widely used in the field of livestock and poultry [7–9]. It has become the preferred technology for researchers to solve various complex biological problems in recent years. Xu et al. [7] studied the Longissimus dorsi muscle of two pig breeds by RNA-seq. The results showed that *ACSL1, FABP3, UCP3* and *PDK4* could be used as candidate genes related to lipid metabolism, and *ASB2, MSTN, ANKRD1* and *ANKRD2* could be used as candidate genes for skeletal muscle growth and development. Zhang et al. [8] studied uterine tissue of 49-week-old Luodao white chickens with low-quality eggshell and normal-quality eggshell and a total of 889 DEGs were detected. Pathway analysis showed that these genes were mainly related to calcium transport and calcium signaling pathway. Ren et al. [9] collected 6-week-old pectoral muscle of slow-growing (Gushi, GS) and fast-growing (Arbor Acres, AA) chicken breeds for RNA-seq. They screened 4815 differentially expressed lncRNAs, identifying two muscle-specific lncRNAs (*TCONS_00064133* and *TCONS_00069348)*.

*RAC family small GTPase 2* (*RAC2*) is a member of the small G protein family. The small G protein is a kind of low molecular weight protein, which can catalyze the transformation between GTP and GDP. It is mainly distributed in the cytoplasm or plasma membrane, and can regulate various cellular physiological processes [10]. The small G protein family associated with *rat sarcoma* (*Ras*) contains about 150 proteins, which can be divided into six families: *Rho, Rab, Ras, Ran, Arf* and *Rad* [11]. Among them, *Rho* protein plays an important role in regulating the structure of the cytoskeleton network and the expression of related genes, ultimately regulating many cellular behaviors [12]. The members of *RAC* protein (*RAC1, RAC2, RAC3*) belong to *Rho* family. *RAC* protein family members regulate cellular behavior in many ways and the most important and well-studied pathway is to activate p21-activated kinases (*PAKs*). At present, there are some studies on the mechanism of *RAC1* regulating cells through *PAKs/MAPKs* signaling pathway [13–15]. However, the function of *RAC2* regulating chicken growth and development through the *PAKs/MAPKs* has not been reported.

In our study, an RNA-seq analysis of the breast muscle of male Jinghai yellow chickens at different growth stages was carried out. We selected a key DEG *RAC2* from RNA-seq and explored its function on fibroblasts proliferation. The research will help us lay a theoretical foundation for further understanding the regulation mechanism of muscle growth and development during chicken growth.

## Results

### RNA quality control and mapping

The results of 1% agarose gel electrophoresis for the total RNA were shown in Figure S1. Two clear bands, 28S and 18S, in each sample were displayed, which suggested that the total RNA did not degrade. The detection results of purity, concentration and integrity for total RNA were shown in Table S3. It showed that the concentration of each sample is within the normal range, with high purity and high integrity. In conclusion, the RNA samples could be used for cDNA library construction.

The data quality control results (Table S4) showed that the clean bases of each sample reached above 7.56G (M8F_1). The percentage of the clean base with 99.9% correct recognition rate (Q30) was more than 90.43% (M4F_2) and the GC content in each sample ranged from 53.93 to 55.16%, indicating that there was no base separation. The sequencing data was good and can be used for a series of subsequent data analysis.

In each sample, the proportion of clean reads mapped to the reference genome was more than 73.63% (M4F_3), which has reached the standard of 70%, and other indicators were also within the normal range (Table S5). Figure S2 showed the distribution of clean reads mapped to the reference genome located in exon, intron and intergenic region. The number of clean reads mapped to exon accounted for the highest proportion in each sample, which was consistent with the reality.

### Screening and functional enrichment analysis of DEGs

**Fig. 1:**
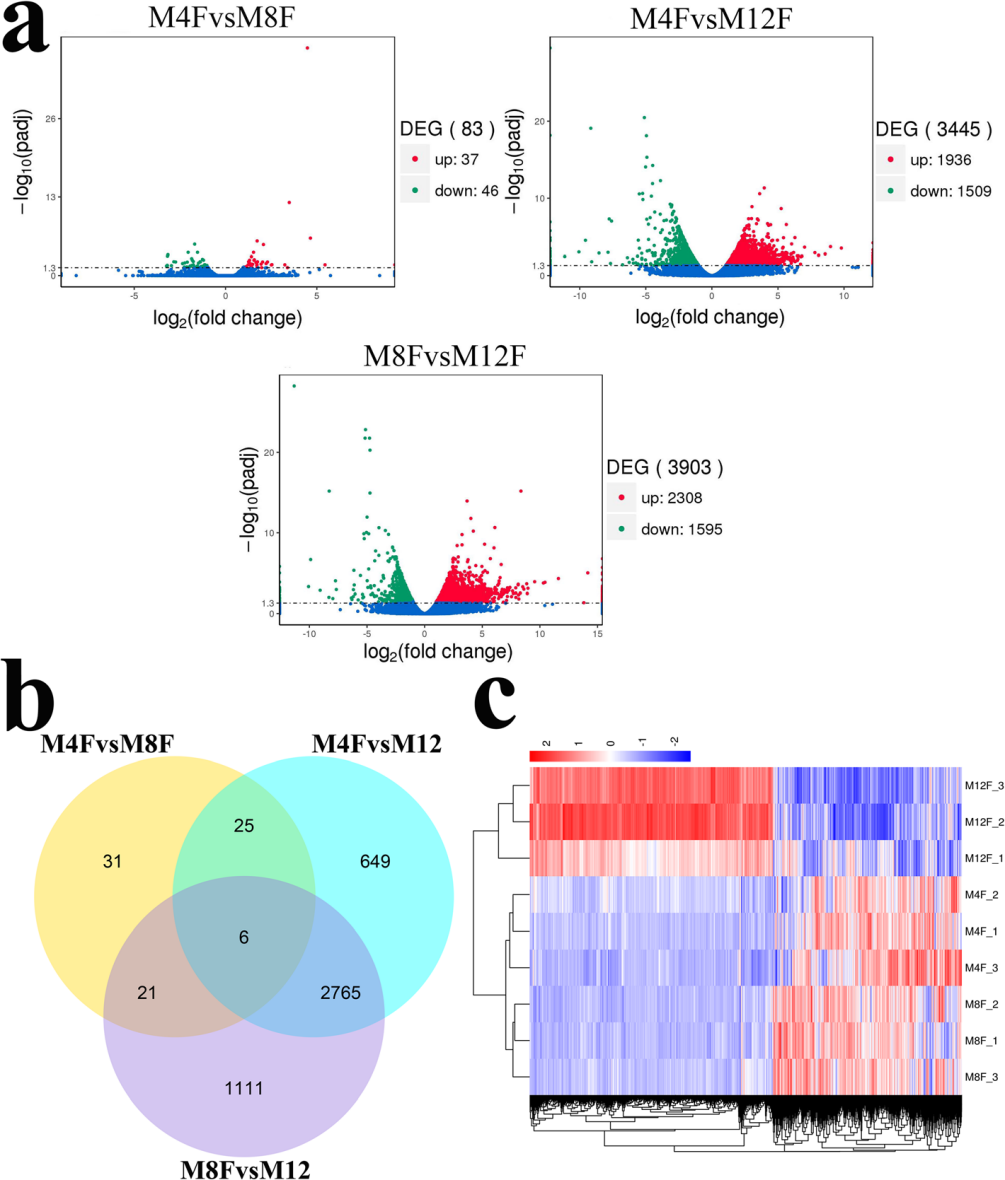
Analysis of differentially expressed genes. **a** The volcano plot of differentially expressed genes. **b** The venn plot of differentially expressed genes. **c** The hierarchical clustering of differentially expressed genes. M4F: Breast muscle of male chickens at 4 weeks; M8F: Breast muscle of male chickens at 8 weeks; M12F: Breast muscle of male chickens at 12 weeks

A total of 4608 DEGs were obtained with the standard FC ≥ 2 and FDR ≤ 0.05 from comparison in pairs of M4F, M8F and M12F. The results showed that 83, 3445 and 3903 DEGs were separately obtained from M4FvsM8F, M4FvsM12F and M8FvsM12F (Fig. 1a). There were 37 up-regulated genes and 46 down-regulated genes in M8F compared with M4F, 1936 up-regulated genes and 1509 down-regulated genes in M12F compared with M4F, and 2308 up-regulated genes and 1595 down-regulated genes in M12F compared M8F. Further analysis showed that six common DEGs were found (Table 1, Fig. 1b), but only three of them were annotated, namely *SNCG*, *MYH1A* and *ARHGDIB*, suggesting that the three genes may be closely related to growth and development at early stages of the Jinghai yellow chicken.

**Table 1:** Co-differentially expressed genes

Ensemble ID	Gene name	Full name of genes
*ENSGALG00000002015*	*SNCG*	*synuclein gamma*
*ENSGALG00000037864*	*MYH1A*	*myosin, heavy chain 1A, skeletal muscle*
*ENSGALG00000011738*	*ARHGDIB*	*Rho GDP dissociation inhibitor beta*
*ENSGALG00000031598*	*–*	*–*
*ENSGALG00000035660*	*–*	*–*
*ENSGALG00000030559*	*–*	*–*

The FPKM of 4608 DEGs was used for hierarchical clustering analysis. The distance between samples was calculated using the expression of DEGs in each sample. The result (Fig. 1c) showed that each sample in the same group was gathered together, which further explained the reliability of the sample selection. The gene expression patterns of breast muscle for chickens were more similar at 4 weeks and 8 weeks. However, it changed greatly at 12 weeks.

**Fig. 2:**
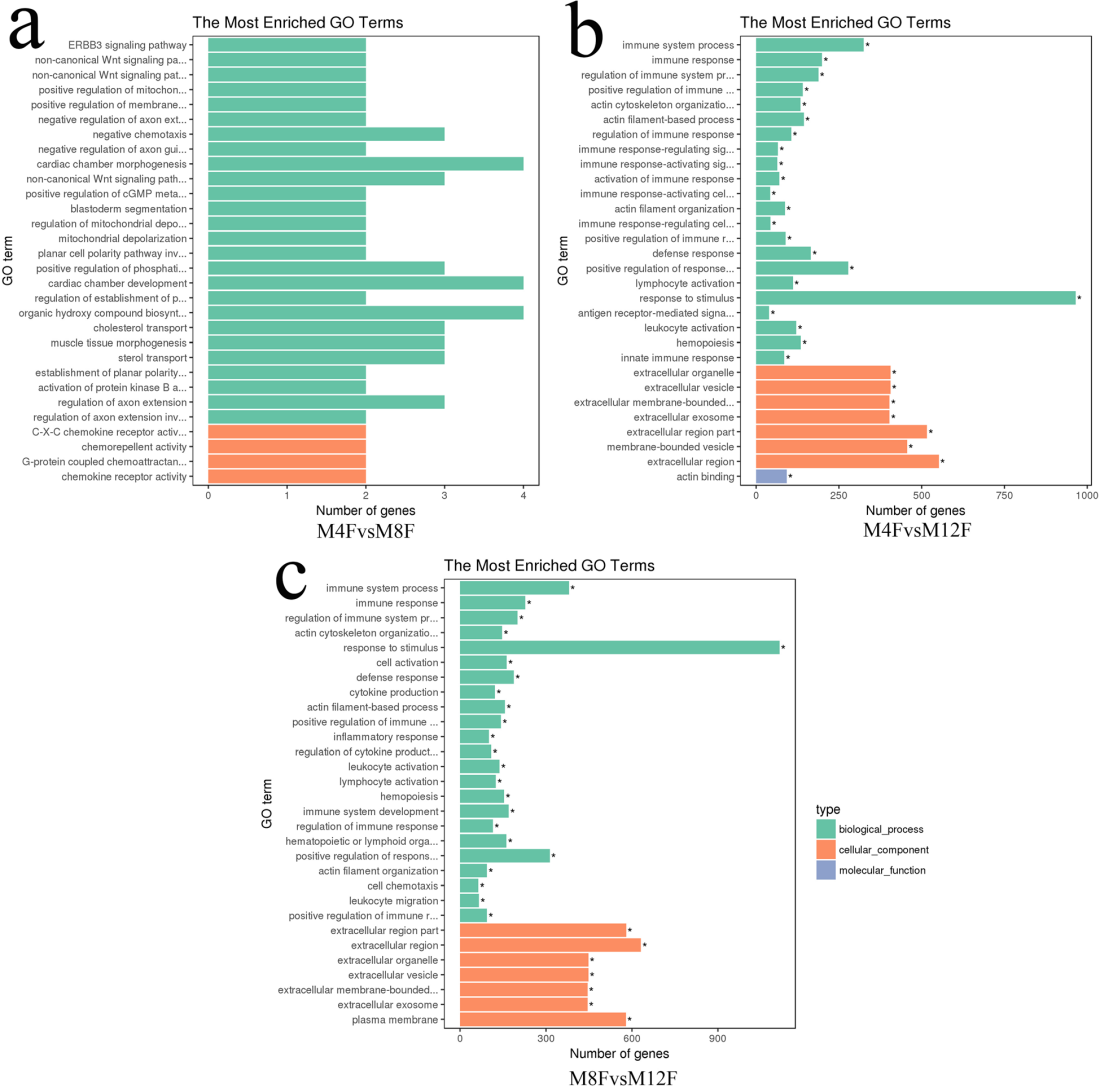
Gene ontology annotation of differentially expressed genes. **a** The top 30 terms in the M4FvsM8F group. **b** The top 30 terms in the M4FvsM12F group. **c** The top 30 terms in the M8FvsM12F group. M4F: Breast muscle of male chickens at 4 weeks; M8F: Breast muscle of male chickens at 8 weeks; M12F: Breast muscle of male chickens at 12 weeks

GO analysis was carried out for 83, 3445 and 3903 DEGs obtained from M4FvsM8F, M4FvsM12F and M8FvsM12F, respectively. Focusing on the biological processes (BP), 304 and 408 terms (corrected p-value < 0.05) were found for M4FvsM12F and M8FvsM12F groups, and no significant BP terms were found for the M4FvsM8F group. Figure 2 showed the histograms for the top 30 terms in the three comparison groups, respectively. The results revealed that no significant enrichment term was found in the M4FvsM8F group (Fig. 2a), which may be due to the small number of DEGs. In the two comparison groups M4FvsM12F and M8FvsM12F, several significantly enriched BP terms (Fig. 2b and c) were obtained and some of them were related to growth and development of chicken (Table 2), including muscle structure development, actin cytoskeleton organization, cell proliferation, cell proliferation, cellular developmental process and cytokine production, etc.

**Table 2:** The biological process related to growth and development

Group	GO accession	Description	Correctedp-value	DEGs number	Growth related genes
M4FvsM12F	GO:0030036	actin cytoskeleton organization	4.66E-10	134	*ACTA1; FGF7; MYL1; TGFB1; MYH9; MEF2A*
	GO:0001816	cytokine production	7.94E-07	102	*TGFB1; MAPK11; IRF4; PRKCD; IRF8*
	GO:0051493	regulation of cytoskeleton organization	3.02E-06	91	*FGF13; TGFB1; MYL1; PAK3; KIF18A; ACTN2*
	GO:0007010	cytoskeleton organization	1.26E-05	215	*FGF13; FGF7; TGFB1; ACTA1; MYH9; MYL1*
	GO:0008283	cell proliferation	0.000199	249	*MYDGF; ING5; FGF7; TGFB1; IGF2; NGFR; IGFBP4; IGF2BP1*
	GO:0007165	signal transduction	0.000229	643	*WNT5B; MEF2C; MEF2A; GDF8; GDF3; GFI1; MYDGF; ING5; FGF13; IGFBP4*
	GO:0003012	muscle system process	0.00951	59	*MYL1; MYLK2; FGF13; TNNT3; TNNC2; MEF2C; MSTN*
	GO:0048869	cellular developmental process	0.023947	477	*GDF3; GAS1; TGFB1; IGF2BP1; GFI1; ACTA1; MYH9; WNT9A; WNT5B*
	GO:0061061	muscle structure development	0.034591	89	*TGFB1; MYH9; ACTA1; BTG1; MEF2C; MEF2A; MEF2D; MSTN*
M8FvsM12F	GO:0001816	cytokine production	2.39E-09	122	*TGFB3; TGFBR2; TGFB1; WNT5A; TGFB3*
	GO:0030036	actin cytoskeleton organization	1.22E-09	147	*MYH9; ACTN1; ARHGAP35; MEF2A; TGFB1; FGF7*
	GO:0008283	cell proliferation	6.81E-06	290	*MSTN; MEF2C; TGFB1; TGFBR2; WNT2; WNT5A; WNT9A; IGF2; IGFBP4; IGF2BP1*
	GO:0032956	regulation of actin cytoskeleton organization	1.35E-05	72	*TGFB3; TGFB1; TGFBR2; ACTN2; MEF2C; MYL1;*
	GO:0048731	system development	0.008234	588	*MSTN; IGF2BP1; MEF2A; MEF2C; MEF2D; WNT5A; WNT2; WNT9A; MYH9*
	GO:0048513	organ development	0.004239	445	*MSTN; WNT2; WNT9A; WNT16; IGF2BP1; MEF2A; MEF2C; MYH9*
	GO:0051094	positive regulation of developmental process	0.02081	189	*WNT9A; TGFBR2; TGFB1; TGFB3; WNT5A; IGF2BP1; MEF2C;*
	GO:0032502	developmental process	0.026142	756	*MSTN; GDF3; MEF2A; MEF2C; MEF2D; MYH9; MYL2K; TGFB1; TGFB3; TGFBR3*
	GO:0061061	muscle structure development	0.041363	99	*WNT2; TGFB1; MEF2A; WNT5A; MEF2D; MSTN; MYH9; ACTN1*
	GO:0048869	cellular developmental process	0.042022	539	*MSTN; GDF3; IGF2BP1; TGFB1; TGFB3; TGFBR2; MYH9*

Note:M4F: Breast muscle of male chickens at 4 weeks; M8F: Breast muscle of male chickens at 8 weeks; M12F: Breast muscle of male chickens at 12 weeks

**Fig. 3:**
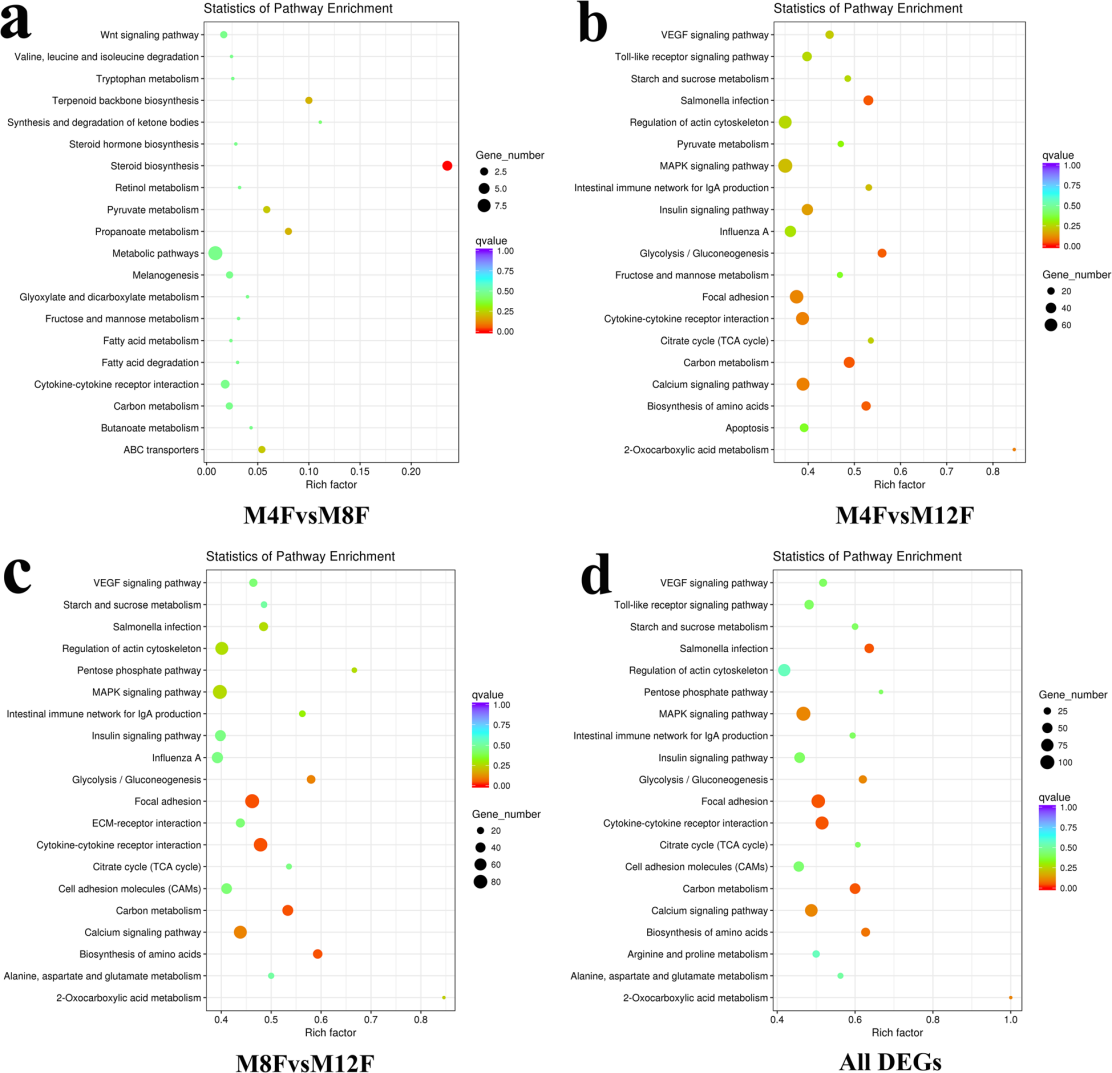
Kyoto Encyclopedia of Genes and Genomes pathway analysis of differentially expressed genes. **a** The top 20 pathways for differentially expressed genes in the M4FvsM8F group. **b** The top 20 pathways in the M4FvsM12F group. **c** The top 20 pathways in the M8FvsM12F group. (d) The top 20 pathways for the all differentially expressed genes. M4F: Breast muscle of male chickens at 4 weeks; M8F: Breast muscle of male chickens at 8 weeks; M12F: Breast muscle of male chickens at 12 weeks

KEGG pathway analysis was performed for the DEGs. The results (Fig. 3, Table 3) showed that 1, 2, 4 and 4 pathways were significantly enriched (corrected *p*-value ≤0.05) in groups M4FvsM8F (Fig. 3a), M4FvsM12F (Fig. 3b), M8FvsM12F (Fig. 3c) and all the DEGs (Fig. 3d), respectively. They are steroid biosynthesis, carbon metabolism, focal adhesion, cytokine-cytokine receptor interaction, biosynthesis of amino acids and salmonella infection. The first five pathways were all closely related to chicken growth and development, and had important reference value in scientific research and production.

**Table 3:** Significant enrichment of KEGG pathways associated with growth and development

Group	Name of the KEGG	Term ID	DEGs number	Corrected p-value	Genes name
M4FvsM8F	Steroid biosynthesis	gga00100	4	0.000391	*NSDHL; SQLE; SC5D; HSD17B7*
M4FvsM12F	Carbon metabolism	gga01200	44	0.049506	*PFKM; HK2; FBP1; FBP2; PKM; MDH2; DLAT*
	Salmonella infection	gga05132	35	0.049506	*MAPK11; FOS; FOSB; IL8L1; RHOG*
M8FvsM12F	Cytokine-cytokine receptor interaction	gga04060	79	0.041333	*TGFB1; TGFB3; TGFBR2; BMPR2; GSF1R; VEGFA; EGF; CCR5*
	Focal adhesion	gga04510	85	0.041333	*MYLK2; ACTN1; ACTN2; MAPK9; RAC2; VAV2; MYLK4; HGF*
	Carbon metabolism	gga01200	48	0.043003	*PKM; FBP1; FBP2; HK1; CPS1; GOT2; GPI*
	Biosynthesis of amino acids	gga01230	35	0.043003	*PKM; PFKM; TKT; MAT1A; CTH*
All DEGs	Carbon metabolism	gga01200	54	0.04593	*PKM; HK1; HK2; HK3; DLAT*
	Focal adhesion	gga04510	93	0.04593	*FN1; PIK3CB; MYLK4; ACTN2; RAC2*
	Cytokine-cytokine receptor interaction	gga04060	85	0.04593	*TGFB1; FAS; BMPR2; TGFB3; TGFBR2;*
	Salmonella infection	gga05132	42	0.04593	*MAPK9; MAPK11; FOSB; IL8L1*

Note:M4F: Breast muscle of male chickens at 4 weeks; M8F: Breast muscle of male chickens at 8 weeks; M12F: Breast muscle of male chickens at 12 weeks

### Validation of RNA-seq results using qPCR

**Fig. 4:**
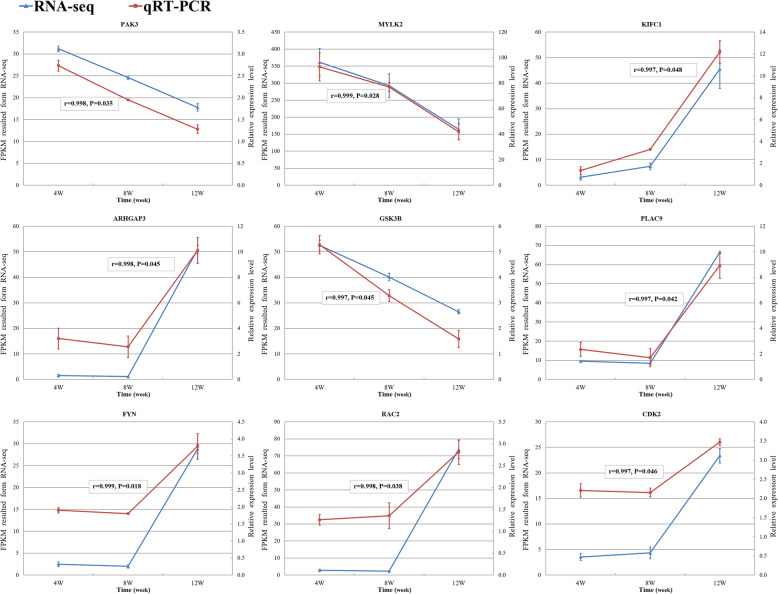
The validation of differentially expressed genes by qPCR

Nine genes were selected for qPCR to verify the accuracy of RNA-seq (Fig. 4). Pearson correlation coefficient (r) was used as the criterion and the significance analysis (P) was also carried out. The results showed that expression of the nine genes were significantly correlated between the RNA-seq and qPCR (r ≥ 0.95 and *P* ≤ 0.05).

### Construction of PPI protein interaction network

**Fig. 5:**
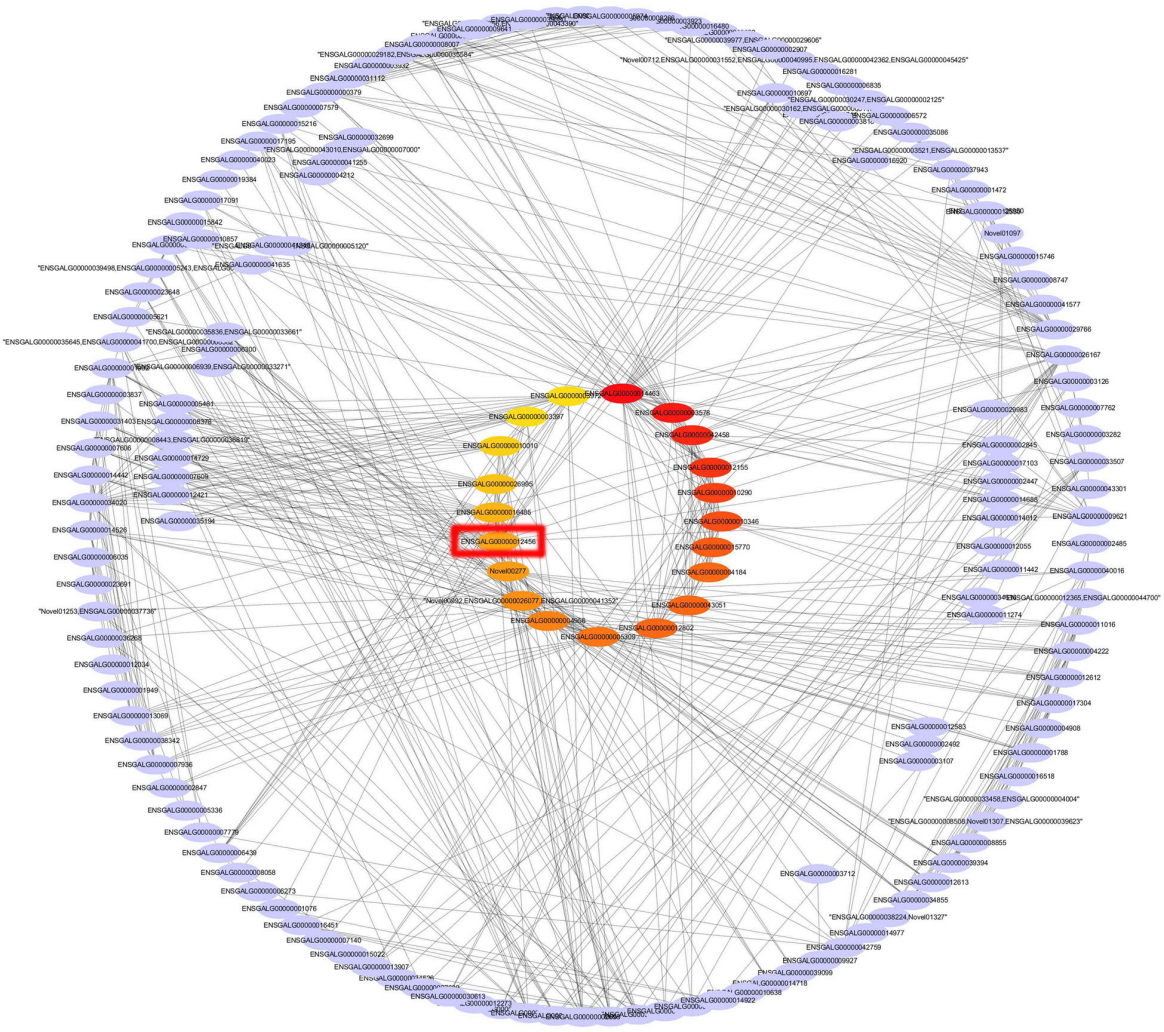
Protein-protein interaction networks

The result of RNA-seq showed that a total of 4608 DEGs were obtained in the three comparison groups. We extracted 2143 pairs of interaction relationships from the STRING database. Finally, the plug-in cytohubba of Cytoscape was used to screen the first 20 hub genes and visualize the network (Fig. 5).

**Fig. 6:**
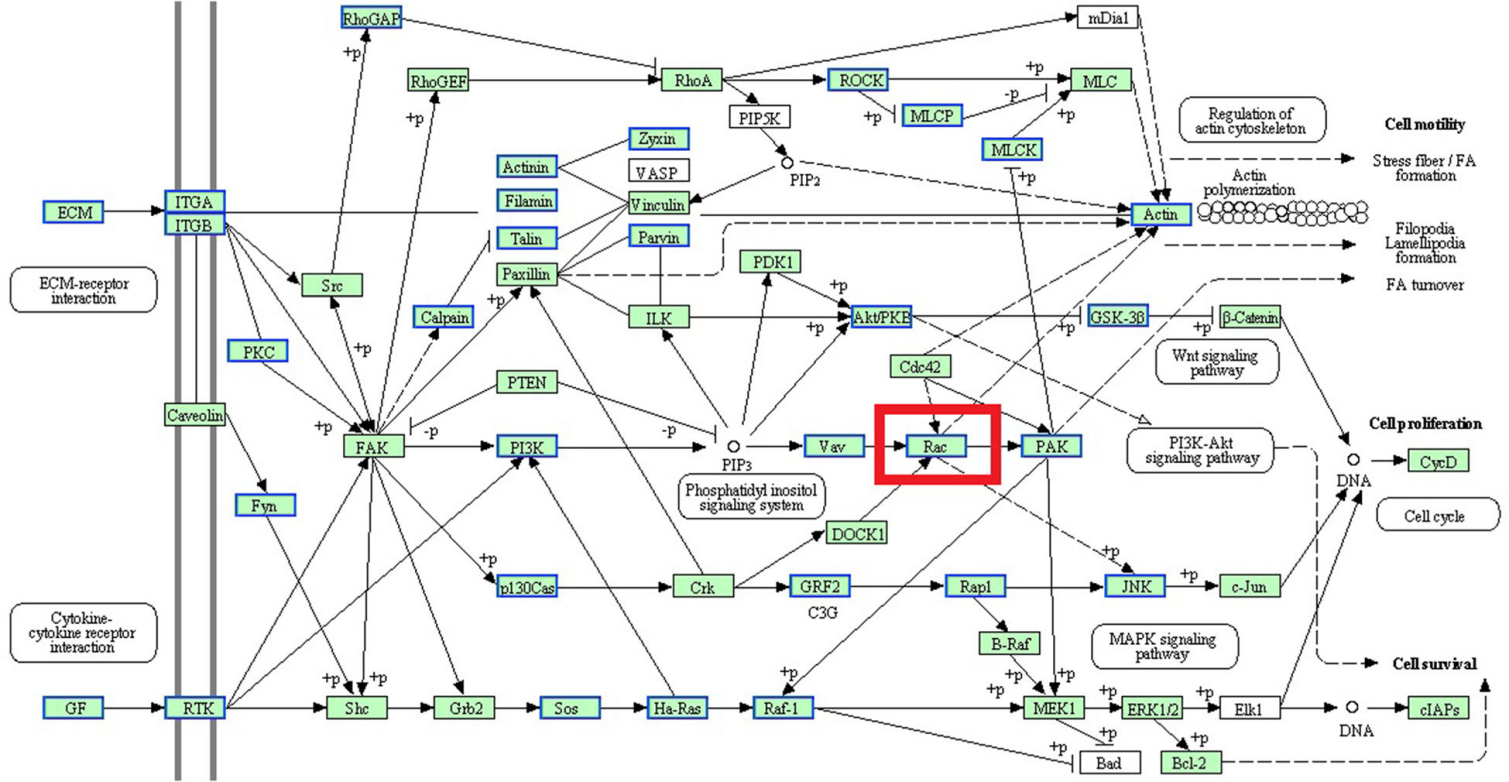
The focal adhesion pathway and I have obtained the copyright permission to use the pathway

Through further analysis, we found that 6 of the 20 hub genes were enriched in focal adhesion pathway, accounting for the highest proportion of the hub genes (Table S6). The focal adhesion pathway was one of the 6 significantly enriched pathways of 4608 DEGs and it is closely related to growth and development [16–18]. *RAC2*, one of the 6 genes enriched in focal adhesion, was at the core of the pathway (Fig. 6). Therefore, *RAC2* was selected as a key gene for cell level functional verification.

### *RAC2* is involved in the *PAKs/MAPK8* pathway

**Fig. 7:**
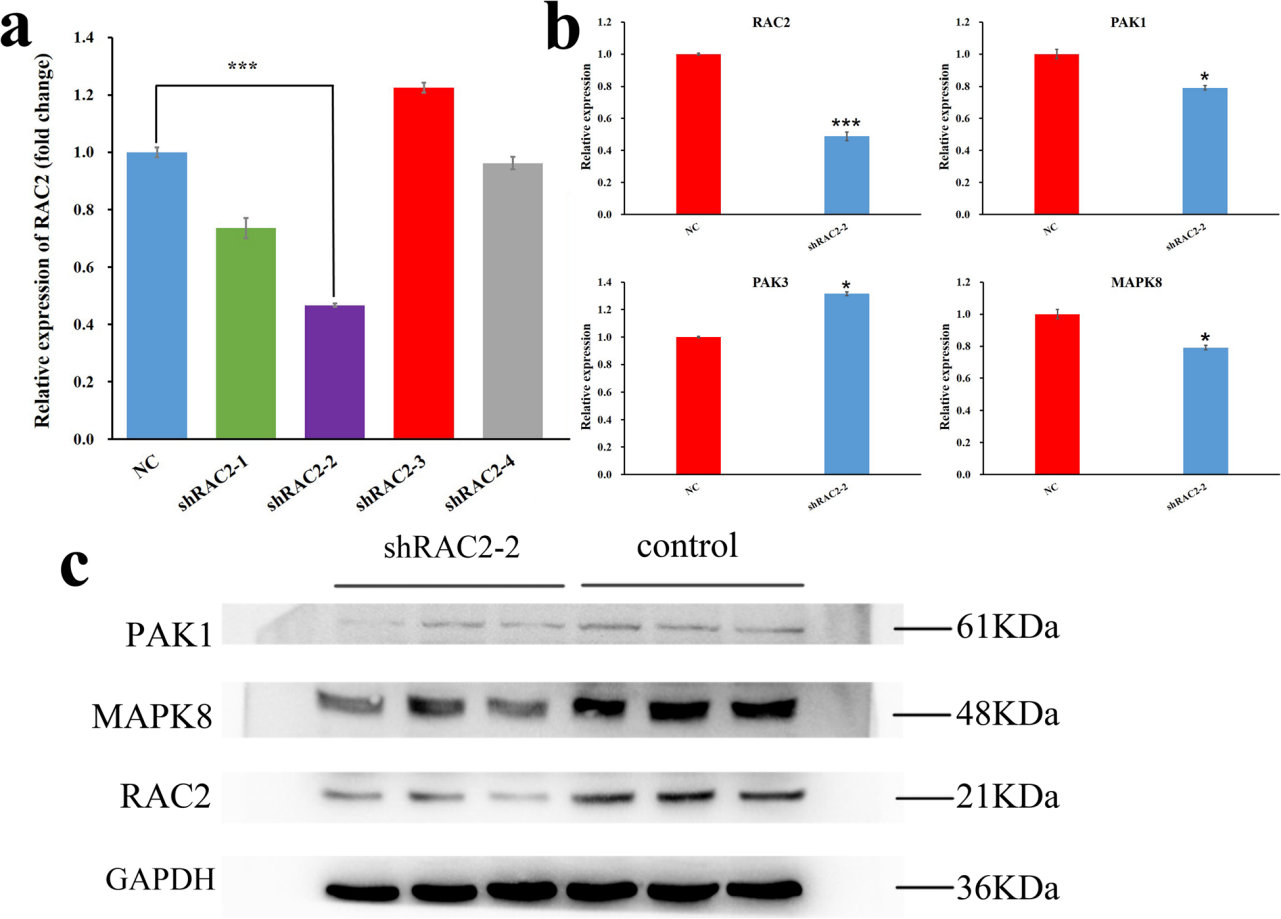
The results of qPCR and western blot. **a** The expression of *RAC2* for the four interference plasmids. **b** Relative expression of mRNA for genes. **c** The protein expression of *RAC2*, *PAK1* and *MAPK8*. The full-length blots are presented in supplementary Fig. S3. *P ≤ 0.05, ***P ≤ 0.001

The four interfering recombinant plasmids of *RAC2* (sh*RAC2*–1, 2, 3 and 4) were transfected into DF-1 cells. The expression of *RAC2* was decreased in four groups (Fig. 7a) compared with the negative control group (NC), among which, the group of sh*RAC2*–2 had the highest interference efficiency of about 60%. The expression of *RAC2* was also decreased significantly (*P* ≤ 0.001) in sh*RAC2*–2 group. Therefore, sh*RAC2*–2 was chosen as an effective interference plasmid for functional verification.

The sh*RAC2*–2 and NC plasmids were transfected into DF-1 cells cultured in 6-well plates, respectively. Total RNA was extracted 24 h after transfection and protein was extracted 48 h after transfection. Quantitative polymerase chain reaction (qPCR) showed that the expression of *RAC2* and its downstream genes *PAK1* and *MAPK8* were significantly decreased (*P* = 0.00, *P* = 0.003 and *P* = 0.032) in the sh*RAC2*–2 group (Fig. 7b). However, the gene *PAK3* was significantly increased (*P* = 0.011) in the sh*RAC2*–2 group (Fig. 7b). Western Blot (WB) also showed that the protein expression of *RAC2*, *PAK1* and MPAK8 were decreased in the sh*RAC2*–2 group (Fig. 7c) and the full-length blots are presented in supplementary Figure S3. All the above results indicated that *RAC2* may affect the proliferation of DF-1 cells through *PAK1/PAK3/MAPK8* pathway.

### *RAC2* promotes the proliferation of fibroblasts

**Fig. 8:**
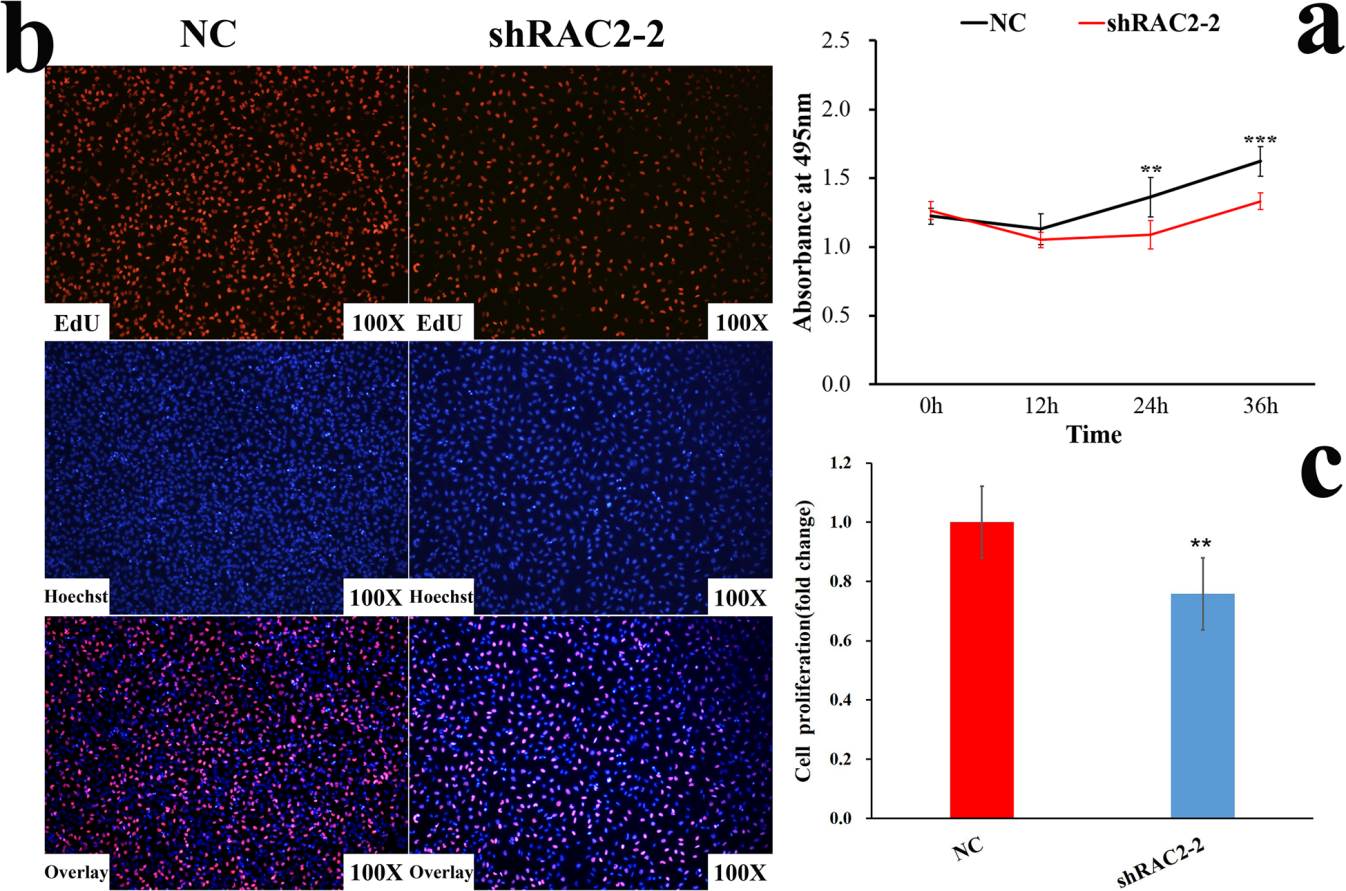
Cell proliferation detection. **a** The absorbance of DF-1 cells for CCK-8 assay. **b** EdU staining of DF-1 cells. (c) Statistical results of cell proliferation proportion of EdU for DF-1 cells. **P ≤ 0.01, ***P ≤ 0.001

The sh*RAC2*–2 and NC plasmids were transfected into DF-1 cells cultured in 96-well plates. The results of cell proliferation using CCK-8 showed that there was no significant difference in absorbance between the sh*RAC2*–2 and NC groups at 0 and 12 h. The absorbance of sh*RAC2*–2 was decreased significantly at 24 and 36 h (Fig. 8a). The results of EdU also showed that the proportion of proliferative cells in sh*RAC2*–2 group was significantly lower (*P* = 0.005) than that of the NC group (Fig. 8b and c). It indicates that *RAC2* can affect the proliferation of DF-1 cells.

## Discussion

Transcriptome sequencing can be used to study the regulation of broilers’ growth and development systematically, and some achievements have also been made in recent years. Chen et al. [17] revealed *FoxO3* as a candidate gene for growth and development for chickens when studying the breast muscle of females of two breeds with divergent growth speed, using RNA-seq. Piorkowska et al. [19] collected the pectoral muscles of eight little hens, and divided them into two groups according to the different shear force. They found several genes related to muscle tenderness, including the *ASB2, THRSP* and *PLIN1*. Li et al. [20] collected breast muscle of female Gushi chickens at 6, 14, 22, and 30 weeks for RNA-seq, and found 388 known miRNAs and 31 novel miRNAs between developmental stages. Li et al. [21] also performed RNA-Seq analysis of breast muscle from female Gushi chickens at two physiological stages, including juvenile (G20W) and laying (G55W). The authors identified 186 lncRNAs and 881 mRNAs differentially expressed between the two stages, mainly involved in ECM-receptor interaction, glycerophospholipid metabolism, ubiquitin-mediated proteolysis, and the biosynthesis of amino acids.

In the current study, we collected the breast muscle of male Jinghai yellow chickens at 4, 8 and 12 weeks for RNA-seq and found some growth related genes of male chicken. Common differentially expressed genes *SNCG* and *ARHGDIB* were mostly found to be related to cancer, and they can regulate cell proliferation, differentiation, invasion, metastasis and other cell behaviors [22–25]. So we speculated that the two genes may regulate the growth and development of chicken by influencing cell behaviors.

Differentially expressed gene *MYH1A* is a member of myosin heavy chain (*MyHC*) family. Myosin, consisting of two heavy chains (*MyHC*) and four light chains (*MyLC*) [26], is the largest component of muscle contractile tissue [27]. The *MyHC* subtype mainly determines the types of muscle fiber [28]. In general, muscle fiber types can be categorized as slow-twitch oxidative (Type I), fast-twitch oxidative/glycolytic (Type IIa), and fast-twitch glycolytic fibers (Type IIb and IIx) [29]. At birth, muscle is composed of oxidative fibers and the proportion of oxidative fibers decreases while the proportion of glycolytic fibers increases during growth [30]. The type of muscle fiber is closely related to the meat quality, because it fundamentally determines the pH, tenderness, and intramuscular fat of muscle [31]. The growth process of poultry, especially the early growth stage, is an important stage in the transformation of muscle fiber types [32, 33]. In the study, RNA-seq showed that the expression of *MYH1A* was significantly decreased at 8 and 12 weeks compared with 4 weeks. In addition, other *MyHC* subtypes such as *MYH1B, MYH1E, MYH1F, MYH1G* and *MYH9* were also differentially expressed among groups. The expression changes of *MyHC* subtypes may reflect the transformation of muscle fiber types and then affect the early growth and development of Jinghai yellow chicken.

Myosin light chain (*MyLC*) can be further divided into essential myosin light chain (*ELC*) and regulatory myosin light chain (*RLC*), which are encoded by a variety of genes, including *MYL1, MYL2, MYL3*, etc. [34]. It is generally believed that the main function of *ELC* is to maintain the configuration of heavy chain, while *RLC* plays a role in regulating the activity of muscle fiber, and the proportion of different types for myosin light chains would affect the type and growth of muscle fiber [35]. In this experiment, we found that the expression of *MYL1* had no significant change between 4 and 8 weeks, but significantly decreased at 12 weeks (FDR ≤ 0.01). The expression of *MYL6* also did not change significantly between 4 and 8 weeks, but increased significantly at 12 weeks (FDR ≤ 0.01). Changes in the expression of these genes indicated that the proportion of muscle fiber types might be different at different growth stages.

*Myocyte enhancer factor 2 (MEF2)* belongs to the MADS-box transcription factor family and it has four members (*MEF2A, MEF2B, MEF2C* and *MEF2D*) [36, 37]. The activation of genes related to skeletal muscle growth and development were regulated by muscle specific transcription factors such as myogenic regulatory factors (*MRFs*), which have a homologous structure of bHLH (basic helix loop helix) [38]. The MADS-box region of *MEF2* can interact with bHLH to activate the expression of skeletal muscle specific genes, and finally regulate the skeletal muscle growth and development [39]. In the study, the expression trends of the three DEGs (*MEF2A, MEF2C* and *MEF2D*) were consistent in different stages of growth, suggesting that they may play the same role in regulating the growth and development of chicken skeletal muscle.

Focal adhesion is a special structure in cells, and it is closely related to growth and development [16–18]. Using the plug-in cytohubba of Cytoscape, we found that 6 of 20 hub genes were enriched in the focal adhesion pathway, accounting for the highest proportion of hub genes. Among them, *RAC2* was at the core of the pathway. Studies have shown that there were three subtypes of *RAC* (*RAC1, RAC2* and *RAC3*), and they have high homology [40]. Some experiments have also shown that the level of active *RAC1* was increased in *RAC2*-deficient cells [41, 42]. We speculated that *RAC2* and *RAC1* have some overlapping functions. *P21-activated kinase* (*PAK*) is an important effector of protein *RAC* [43, 44]. *PAKs* are classified into two categories: *PAK I* and *PAK II* [45]. *PAK I* includes *PAK1*, *PAK2* and *PAK3* [46]. *PAKs* are involved in various biological effects, such as cytoskeleton recombination, cell migration, cell proliferation and gene transcription [47–49]. Members of the *Mitogen-activated Protein Kinases* (*MAPKs*) superfamily are important downstream signaling molecules of *PAKs* [50, 51]. *MAPKs* are abundant in eukaryotic cells, which can transmit extracellular signals to the nucleus and regulate various physiological and biochemical processes in cells [52]. As a member of *MAPK* signaling pathway, *MAPK8*, also known as *JNK, JNK1* and *SAPK1*, participates in many biological and molecular processes, such as cell proliferation, differentiation and apoptosis [53–55]. Wang et al. [56] found that *MAPK8* could promote the differentiation of chicken embryonic stem cells into spermatogonial stem cells.

In the study, we also explored the function of *RAC2* on fibroblasts proliferation through the *PAKs/MAPKs* signaling pathway. Results of qPCR showed that the expression of *RAC2* was significantly lower than that of the NC group, and the expression of *PAK1* was also significantly lower, which was consistent with the expression trend between *RAC1* and *PAK1* in other species [57–59]. The protein expression of *RAC2* and *PAK1* were also decreased in the sh*RAC2*–2 group. However, the expression of *PAK3* was significantly increased, which was consistent with the results of Chen [60]. We infer that *RAC2* may act on *PAKs* in the same way as *RAC1*, while the function of *PAK3* may be opposite to that of *PAK1*. The results of RNAi for *RAC2* showed that the expression of *MAPK8* was significantly lower than that of the NC group. Western Blot showed that *MAPK8* protein was also decreased. Finally, CCK-8 and EdU assays both showed that proliferation of DF-1 cells was inhibited after transfection of sh*RAC2*–2. Therefore, it could be inferred that knockdown of *RAC2* inhibits the proliferation of DF-1 cells by down-regulating *PAK1* and *MAPK8* while up-regulating *PAK3*.

## Conclusions

The study systematically revealed genes, BP terms and KEGG pathways related to growth and development of male Jinghai yellow chickens. It will lay a theoretical foundation for uncovering the molecular mechanism of skeletal muscle growth and development, and also has an important guiding role in practical production. Further functional research in DF-1 cells showed that differentially expressed gene *RAC2* might regulate cell proliferation by regulating *PAKs/MAPK8* pathway and affect the growth of chickens.

## Materials

### Animals

The chickens in the experiment were obtained from the same group of Jinghai yellow chicken in Jiangsu Jinghai Poultry Industry Group Co., Ltd. (Nantong, Jiangsu Province, China). At 4 (M4F), 8 (M8F) and 12 (M12F) weeks of age, three healthy male chickens with similar body weight (Table S1) were selected for slaughter, respectively. The chickens were first anesthetized with 8 mg/kg of Xylazine Hydrochloride (SIGMA, X-1251). Then, when the feathers on both wings and tails fell down and failed to stimuli respond, it showed complete anesthesia. Following that, they were all sacrificed with bleeding of the carotid artery. Finally, the left breast muscles were collected and frozen in liquid nitrogen for RNA-seq.

### cDNA library construction and sequencing

Total RNA was isolated using the TRIzol total RNA extraction kit (Invitrogen, Carlsbad, CA, USA). RNA degradation and contamination were monitored on 1% agarose gels. RNA purity was checked using the NanoPhotometer® spectrophotometer (IMPLEN, CA, USA). RNA concentration was measured using Qubit® RNA Assay Kit in Qubit® 2.0 Flurometer (Life Technologies, CA, USA). RNA integrity was assessed using the RNA Nano 6000 Assay Kit on the Bioanalyzer 2100 system (Agilent Technologies, CA, USA). Sequencing libraries were generated using NEBNext® Ultra™ RNA Library Prep Kit for Illumina® (NEB, USA). The cDNA libraries were finally sequenced on the Illumina NovaSeq 5000 platform and 150 bp paired-end reads were generated.

### Data analysis

Raw data (raw reads) of fastq format were firstly processed through in-house perl scripts and clean data (clean reads) were obtained in this step. At the same time, Q30 and GC content of the clean data were calculated. All the downstream analysis was based on the clean data.

We used HISAT (2.0.4) [61] for the reads mapping and HTSeq (v0.6.1) [62] to count the reads numbers mapped to genome. And then Fragments Per Kilobase per Million (FPKM) of each gene was calculated based on the length of the gene and reads count mapped to this gene. Differential expression analysis of the three age groups was performed using the DESeq R package (1.12.0) [63]. The DEGs were finally obtained between groups with the standard Fold Change (FC) ≥ 2 and False Discovery Rate (FDR) ≤ 0.05.

Gene Ontology (GO) enrichment analysis of DEGs was implemented using the GOseq R package (2.12) [64] and the GO terms with corrected *p*-value ≤0.05 were considered significantly enriched [65]. KOBAS (v2.0) [66] was used to analyze the KEGG pathway (corrected p-value ≤0.05) of DEGs.

PPI (protein-protein interactions) analysis of all DEGs was based on the STRING database [67]. We constructed the network by extracting the target gene list from the database. Blastx was used to align the target gene sequences to the selected reference protein sequences, and then the networks were built according to the known interaction of selected reference species. Finally, Cytoscape (3.6.1) was used to visualize the networks, and the plug-in cytohubba was used to find hub genes with the matthews correlation coefficient (MCC) method.

### Validation of the RNA-seq data

Nine DEGs were selected to validate the RNA-seq data and they all have a high level of expression with FPKM > 10. The primers (Table S2) were designed using Primer 5.0 based on the Gallus_gallus-5.0 (NCBI) and synthesized by Sangon Biotech Co., Ltd. (Shanghai, China). B2M [68] was selected as the internal reference in the quantitative polymerase chain reaction (qPCR). The qPCR was implemented using the ChamQ SYBR qPCR Master Mix Kit (Vazyme, Nanjing, China). Three technical replicates were performed for each sample. We calculated the relative expression of genes by 2^-△△Ct^ method and used the SPSS13.0 software to analyze the correlation between qPCR and RNA-seq..

### *RAC2* recombination interference vector screening and function verification

Four shRNAs (short hairpin RNAs) of *RAC2* (Table 4) were designed according to the mRNA sequence (NCBI: NM_001201452.1) on website http://rnaidesigner.thermofisher.com/rnaiexpress/ and they were constructed into the plasmid vector by Gene Create company (Wuhan, China). The four recombinant plasmids were transfected into DF-1 cells, respectively. Expression level of *RAC2* was calculated 24 h after transfection, and the significance test for *RAC2* between interference group (sh*RAC2*–1, 2, 3 and 4) and negative control group was carried out using SPSS13.0 software.

**Table 4:** The oligomeric single stranded DNA sequence of shRNAs

Name	The oligomeric single-stranded DNA sequences
sh*RAC2*–1	GGATCCGCTATACCACTAATGCCTTCCTTCAAGAGAGGAAGGCATTAGTGGTATAGCTTTTTT
sh*RAC2*–2	GGATCCGGATCTTCGTGATGACAAAGATTCAAGAGATCTTTGTCATCACGAAGATCCTTTTTT
sh*RAC2*–3	GGATCCGCTTTCTCCTATTACATATCCTTCAAGAGAGGATATGTAATAGGAGAAAGCTTTTTT
sh*RAC2*–4	GGATCCGCAGTGAGTGAGACCCAATAATTCAAGAGATTATTGGGTCTCACTCACTGCTTTTTT

Note: The underlines are the interference sequences and their complementary sequences

DF-1 cells were collected to detect the mRNA expression of downstream genes 24 h after transfection with shRNAs and detect the protein expression 48 h after transfection. Primers (Table S2) of *RAC2* and its downstream genes for qPCR were also designed using primer 5.0 and synthesized by Sangon Biotech (Shanghai, China) as well.

RIPA (Radio Immunoprecipitation Assay, P0013B) and PMSF (Phenylmethanesulfonyl fluoride, ST506) for the protein extraction were purchased from Beyotime (Shanghai, China). The concentration of the extracted protein was detected using a Bradford Kit (Beyotime, Shanghai, P0006C). The primary antibodies of *RAC2* and *PAK1* were purchased from ABclonal (Wuhan, China) and the dilution ratios were both 1:1000. For MAPK8, the primary and second antibodies were purchased from Sangon Biotech (Shanghai, China), and the dilution ratios were 1:1000 and 1:5000, respectively. For GAPDH, the primary antibody was purchased from HuaBio (Hangzhou, China), and we used a 1:2000 dilution ratio. And all the membranes were cut before staining according to the protein ladder (Thermo Fisher Scientific, MA, USA).

The CCK-8 Cell Counting kit (Vazyme, Nanjing, China) and EdU kit (RIBOBIO, Guangzhou, China) were used to detect cell proliferation in 96-well plates, according to the instructions. The proliferation of CCK-8 was detected 0, 12, 24 and 36 h after transfection. The proliferation of EdU was detected 24 h after transfection. Six biological replicates were set up in the CCK-8 assay. Four replicates were set up in the EdU assay and four visual fields in each replicate were randomly selected for statistical analysis.

### Statistical analysis

The significance of CCK-8, EdU and mRNA expression between sh*RAC2*–2 and NC groups was calculated using SPSS13.0 software with the method Independent-Samples T Test. All data were presented as mean ± SD (standard deviation).

## Supplementary Information

**Additional file 1: Figure S1.** The agarose gel electrophoresis of total RNA.

**Additional file 2: Figure S2.** The distribution of reads in different regions of reference genome.

**Additional file 3: Figure S3.** Original images for western blot.

**Additional file 4: Table S1**. Body weight at different weeks.

**Additional file 5: Table S2.** Primer sequences for qPCR.

**Additional file 6: Table S3.** Quality detection results for RNA.

**Additional file 7: Table S4.** The results of quality control for RNA-seq.

**Additional file 8: Table S5.** The results of comparison with reference genome for clean reads.

**Additional file 9: Table S6.** Information of the top 20 hub genes.

## Data Availability

The raw data can be found in the NCBI BioProject database under the accession number PRJNA661705 (https://www.ncbi.nlm.nih.gov/sra/PRJNA661705). PPI (protein-protein interactions) analysis of all DEGs was based on the STRING database (https://string-db.org/). The shRNAs were designed on the website of Thermo Fisher Scientific (http://rnaidesigner.thermofisher.com/rnaiexpress/). The accession number of *RAC2* is NM_001201452.1 on the website of NCBI (https://www.ncbi.nlm.nih.gov/).

## Abbreviations

DEGs: Differentially expressed genes
FC: Fold Change
FDR: False Discovery Rate
WB: Western Blot
*PAKs*: *p21-activated kinases*
MAPKs: Mitogen-activated Protein Kinases
GO: Gene Ontology
KEGG: Kyoto Encyclopedia of Genes and Genomes
PPI: Protein-protein interactions
MCC: Matthews correlation coefficient
qPCR: Quantitative polymerase chain reaction
CCK-8: Cell Counting Kit-8
EdU: 5-Ethynyl-2’- deoxyuridine
NC: Negative control group
FPKM: Fragments Per Kilobase per Million
MF: Molecular function
BP: Biological process
CC: Cell composition
