# Supplementary figures and images for: Study on the transcriptome for breast muscle of chickens and the function of key gene RAC2 on fibroblasts proliferation
Source: BMC Genomics. 2021 Mar 6;22:157. doi: 10.1186/s12864-021-07453-0 (PMC7937270; doi:10.1186/s12864-021-07453-0)

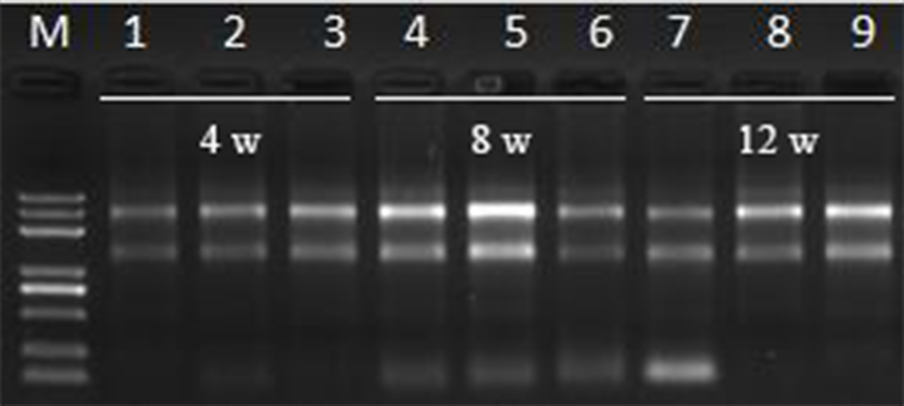

Supplement: Supplementary file 1 — Additional file 1: Figure S1. The agarose gel electrophoresis of total RNA. [file 12864_2021_7453_MOESM1_ESM.tif]

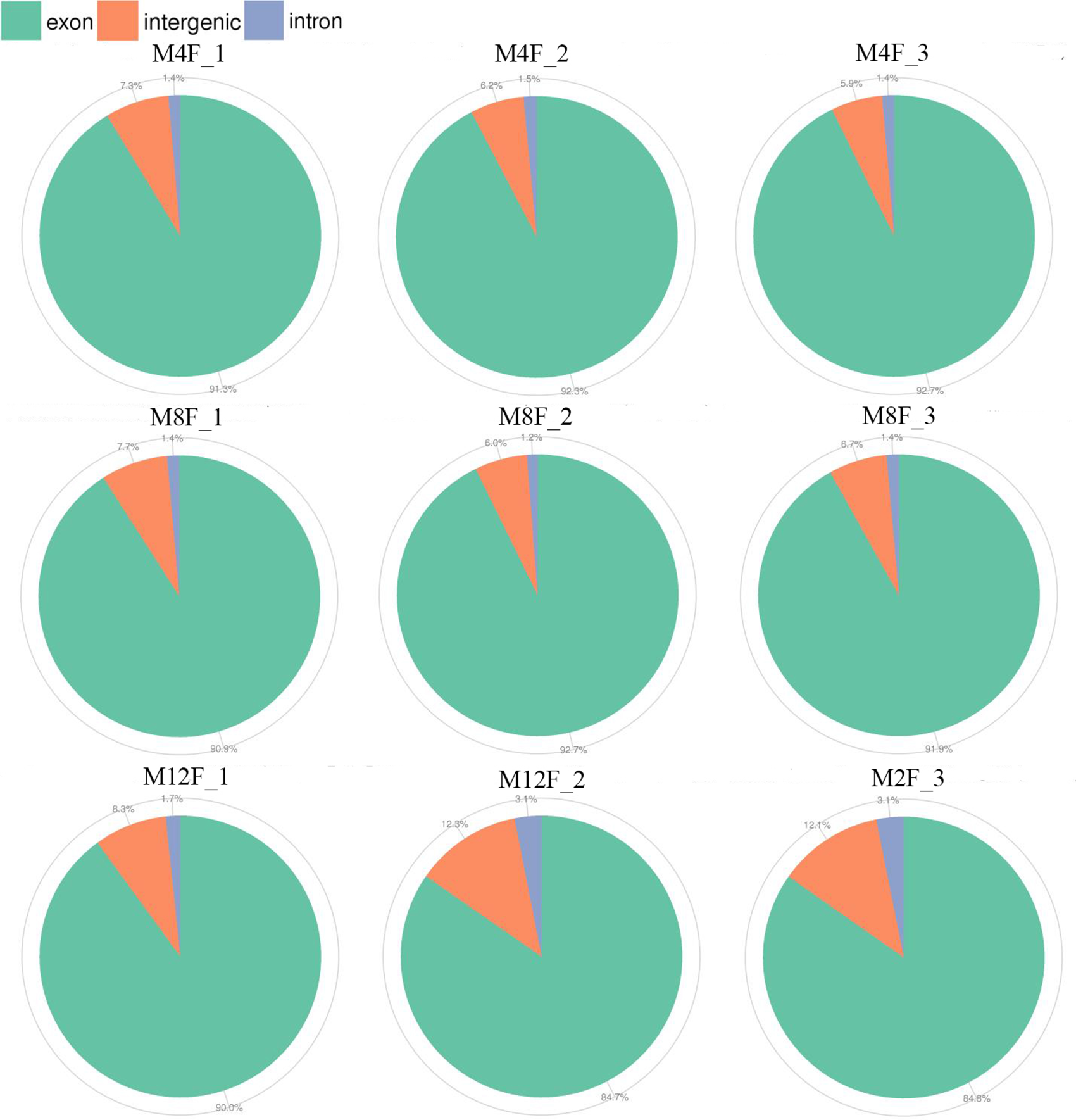

Supplement: Supplementary file 2 — Additional file 2: Figure S2. The distribution of reads in different regions of reference genome. [file 12864_2021_7453_MOESM2_ESM.tif]
