# Supplementary material for: Study on the transcriptome for breast muscle of chickens and the function of key gene RAC2 on fibroblasts proliferation
Source: BMC Genomics. 2021 Mar 6;22:157. doi: 10.1186/s12864-021-07453-0 (PMC7937270; doi:10.1186/s12864-021-07453-0)
Supplement: Supplementary file 3 — Additional file 3: Figure S3. Original images for western blot. [file 12864_2021_7453_MOESM3_ESM.docx]

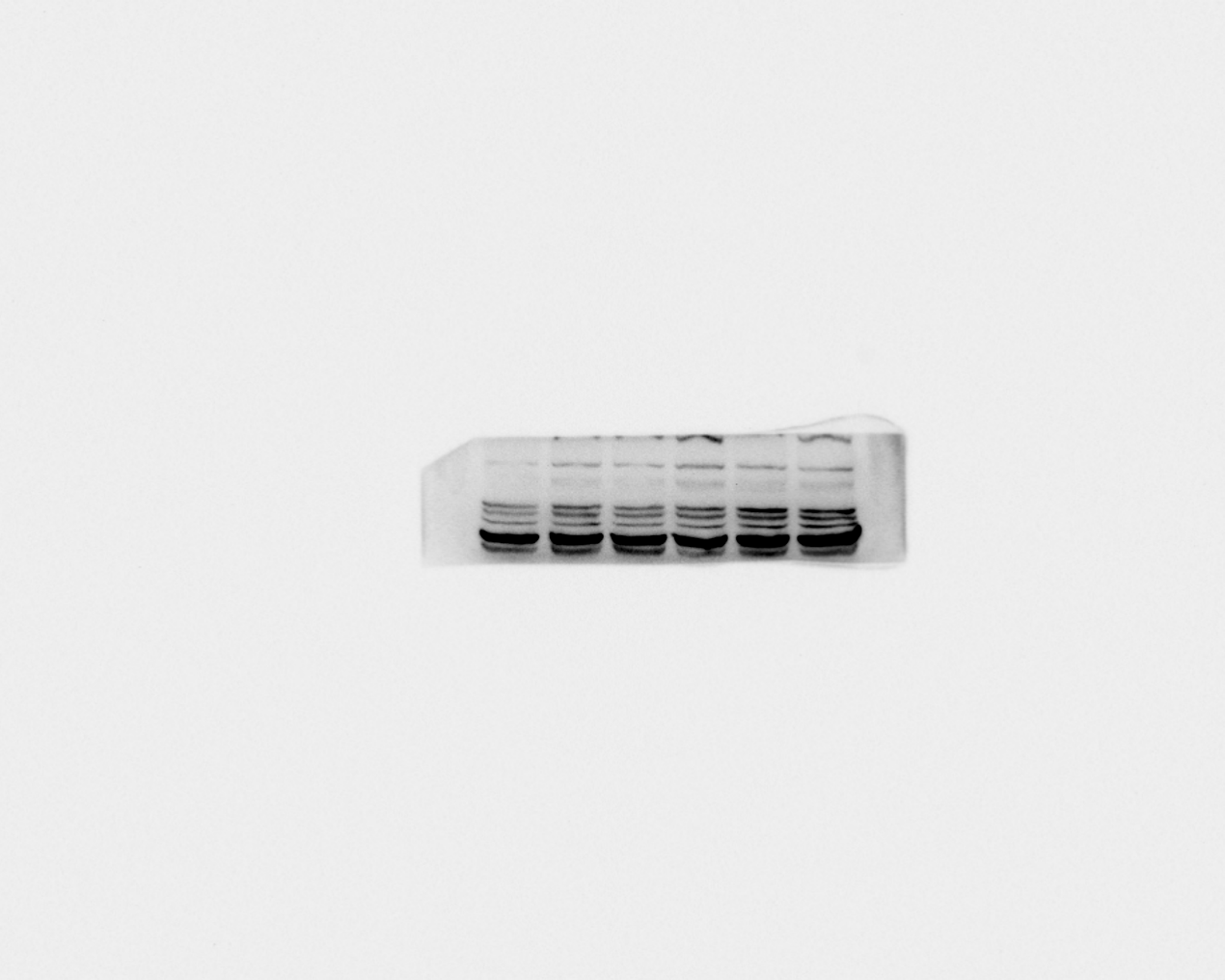


Fig S3-1. Original image of PAK1 for WB and the WB band in the red box is the target band.


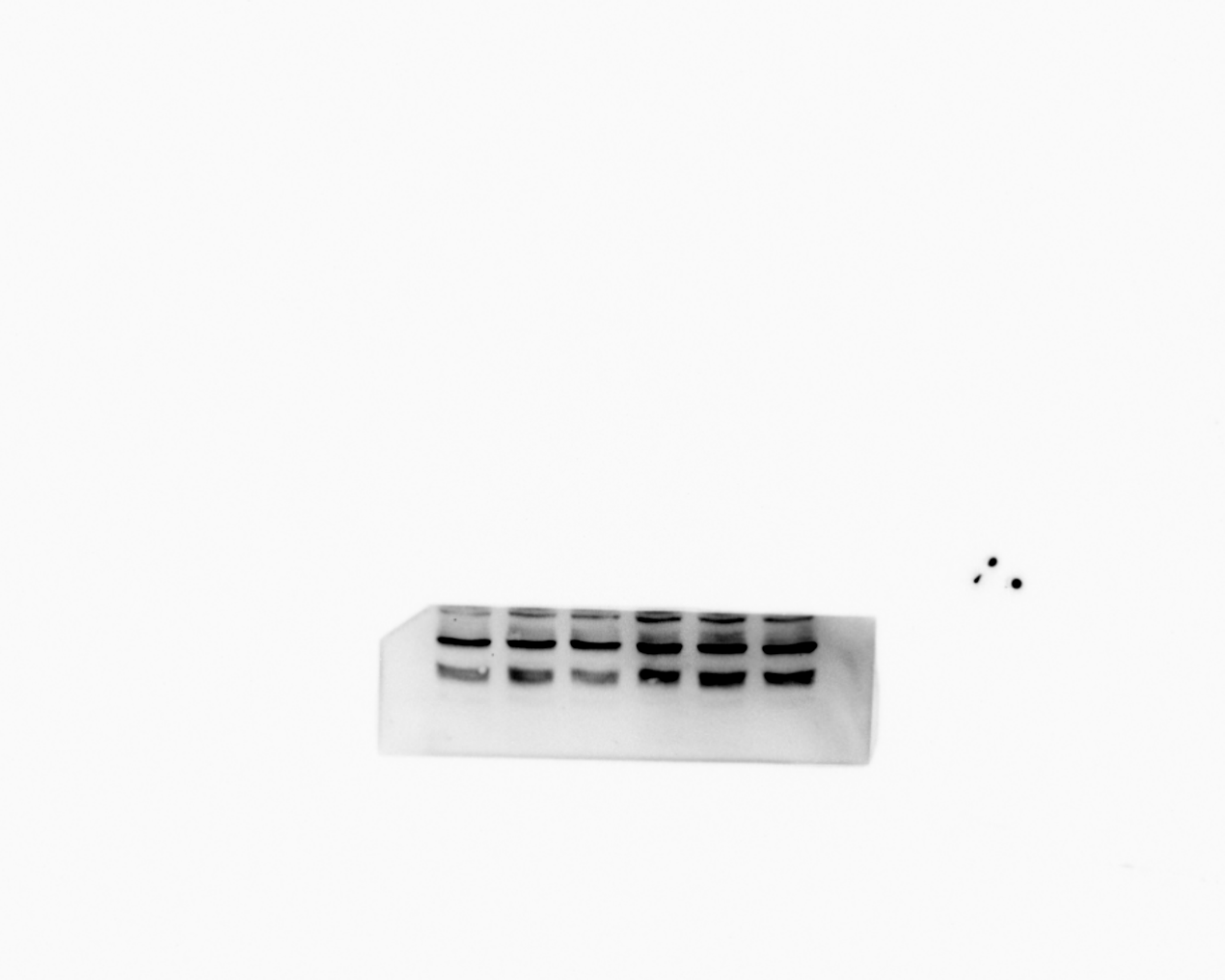


Fig S3-2. Original image of MAPK8 for WB and the WB band in the red box is the target band.


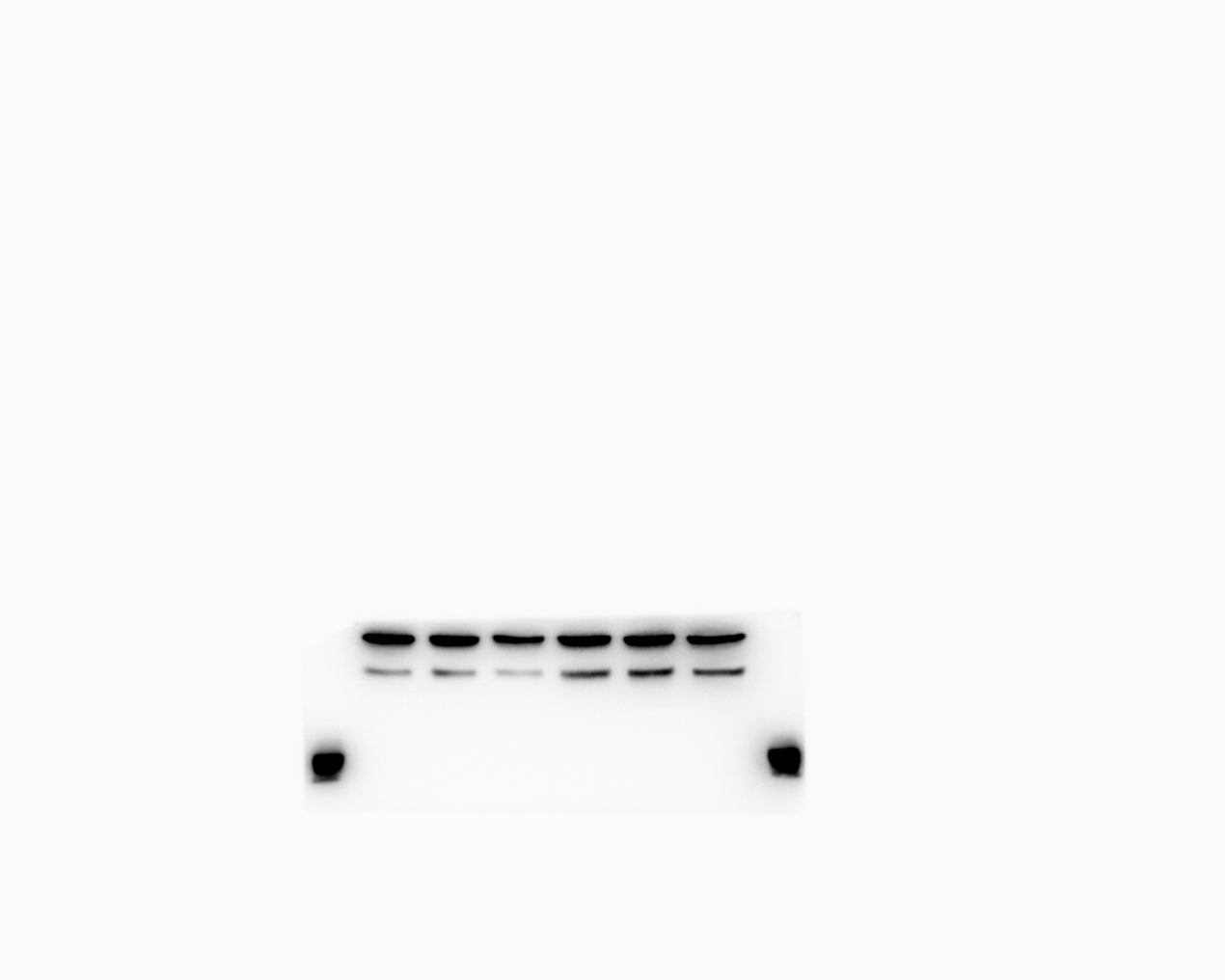


Fig S3- 3. Original image of RAC2 for WB and the WB band in the red box is the target band.


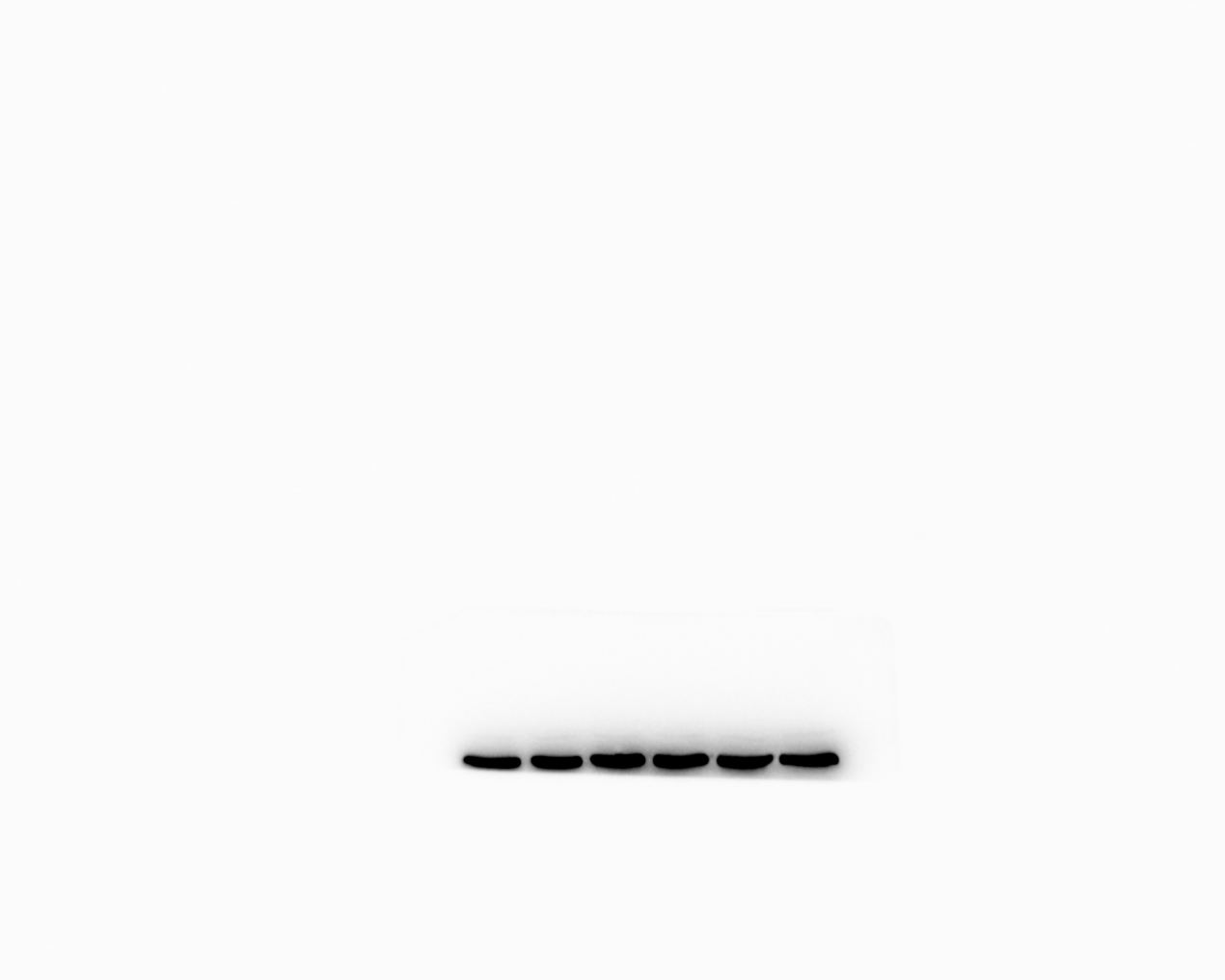


Fig 3-4. Original image of GAPDH for WB
