# Supplementary material for: Study on the transcriptome for breast muscle of chickens and the function of key gene RAC2 on fibroblasts proliferation
Source: BMC Genomics. 2021 Mar 6;22:157. doi: 10.1186/s12864-021-07453-0 (PMC7937270; doi:10.1186/s12864-021-07453-0)
Supplement: Supplementary file 4 — Additional file 4: Table S1. Body weight at different weeks. [file 12864_2021_7453_MOESM4_ESM.docx]

Table S1 Body weight at different weeks

| **Sample name** | Body weight(g) |
| --- | --- |
| M4F_1 | 318 |
| M4F_2 | 295 |
| M4F_3 | 335 |
| M8F_1 | 955 |
| M8F_2 | 910 |
| M8F_3 | 980 |
| M12F_1 | 1210 |
| M12F_2 | 1240 |
| M12F_3 | 1350 |
