# Supplementary material for: Study on the transcriptome for breast muscle of chickens and the function of key gene RAC2 on fibroblasts proliferation
Source: BMC Genomics. 2021 Mar 6;22:157. doi: 10.1186/s12864-021-07453-0 (PMC7937270; doi:10.1186/s12864-021-07453-0)
Supplement: Supplementary file 5 — Additional file 5: Table S2. Primer sequences for qPCR. [file 12864_2021_7453_MOESM5_ESM.docx]

Table S2 Primer Sequences for qPCR

| **Gene name** | **Primer sequence** | **Product（bp）** | **Annealing（°C）** |
| --- | --- | --- | --- |
| B2M | F: GTGCTGGTGACCCTGGTG | 143 | 60 |
|  | R: CAGTTGAGGACGTTCTTGGTG |  |  |
| PAK3 | F: CCCTGAGGAGAAGAACAAG | 151 | 60 |
|  | R: AGTGACGGCATCAAACCC |  |  |
| MYLK2 | F: CAGCCCCAGGTCGGGTCAGA | 204 | 60 |
|  | R: CGTCCGCAGCGTGATGAT |  |  |
| KIFC1 | F: GCACAAAGGTTGGATGGGG | 148 | 60 |
|  | R: ATTCCTGGCGGTGCTTCT |  |  |
| MYH1A | F: ACCAAGCAGCCCAGGCAGTA | 214 | 60 |
|  | R: AGGCAGCCAAGTCCATCCC |  |  |
| GSK3B | F: ACCTCTTGCTGGACCCTG | 126 | 60 |
|  | R: TCAACTCTGGTGCCCTGT |  |  |
| PLAC9 | F: GCTGTTCATCTGGGCACT | 149 | 60 |
|  | R: CCTCAATAATATCTAAGCGTTC |  |  |
| FYN | F: AAGGAGACTGGTGGGAGG | 119 | 60 |
|  | R: GCCAAGTTTGCCGAAGTA |  |  |
| RAC2 | F: TGATGGTTGACAGCAAGCCT | 163 | 60 |
|  | R: AACCACTTAGCACGGACGTT |  |  |
| CDK2 | F: TGCACCAGGACCTGAAGAA | 170 | 60 |
|  | R: ACCGTCGGCGTTGATGAG |  |  |
| GAPDH | F: GTAGTGAAGGCTGCTGCTGATG | 106 | 60 |
|  | R: CAAAGGTGGAGGAATGGCTGTC |  |  |
| PAK1 | F: GTGTGCCGAGAGTGTCTTCAG | 223 | 60 |
|  | R: GCCCGTATGCTTTCCGTGTC |  |  |
| MAPK8 | F:AGAGGGAGCACACAATAGAAGAATG | 165 | 60 |
|  | R:TTGACAGATGACGACGAAGATGG |  |  |
