# Supplementary material for: Study on the transcriptome for breast muscle of chickens and the function of key gene RAC2 on fibroblasts proliferation
Source: BMC Genomics. 2021 Mar 6;22:157. doi: 10.1186/s12864-021-07453-0 (PMC7937270; doi:10.1186/s12864-021-07453-0)
Supplement: Supplementary file 6 — Additional file 6: Table S3. Quality detection results for RNA. [file 12864_2021_7453_MOESM6_ESM.docx]

Table S3 Quality detection results for RNA

| **Sample name** | **Concentration (ng/ul)** | **OD260/280** | **OD260/230** | **28S/18S** | **RNA integrity Number** |
| --- | --- | --- | --- | --- | --- |
| M4F_1 | 70 | 2.059 | 1.944 | 1.4 | 8.5 |
| M4F_2 | 92 | 2.000 | 2.091 | 1.6 | 8.9 |
| M4F_3 | 104 | 2.167 | 1.926 | 1.5 | 8.9 |
| M8F_1 | 94 | 1.880 | 1.880 | 1.3 | 8.7 |
| M8F_2 | 154 | 1.974 | 1.974 | 1.2 | 8.6 |
| M8F_3 | 50 | 1.562 | 1.786 | 1.4 | 8.9 |
| M12F_1 | 176 | 1.833 | 1.114 | 1.0 | 8.1 |
| M12F_2 | 350 | 1.923 | 2.303 | 1.4 | 8.8 |
| M12F_3 | 272 | 1.889 | 2.030 | 1.2 | 7.9 |
