# Supplementary material for: Study on the transcriptome for breast muscle of chickens and the function of key gene RAC2 on fibroblasts proliferation
Source: BMC Genomics. 2021 Mar 6;22:157. doi: 10.1186/s12864-021-07453-0 (PMC7937270; doi:10.1186/s12864-021-07453-0)
Supplement: Supplementary file 7 — Additional file 7: Table S4. The results of quality control for RNA-seq. [file 12864_2021_7453_MOESM7_ESM.docx]

Table S4 The results of quality control for RNA-seq

| **Sample name** | **Raw reads** | **Clean reads** | **Clean bases** | **Q30(%)** | **GC content(%)** |
| --- | --- | --- | --- | --- | --- |
| M4F_1 | 61102148 | 57553102 | 8.63G | 90.66 | 54.20 |
| M4F_2 | 63629812 | 60018588 | 9G | 90.43 | 54.17 |
| M4F_3 | 62940690 | 59343986 | 8.9G | 90.56 | 54.90 |
| M8F_1 | 52721010 | 50382466 | 7.56G | 92.54 | 54.03 |
| M8F_2 | 57894528 | 54856958 | 8.23G | 92.21 | 54.64 |
| M8F_3 | 62623374 | 59583598 | 8.94G | 92.19 | 54.04 |
| M12F_1 | 58195248 | 55942770 | 8.39G | 92.21 | 53.93 |
| M12F_2 | 67436352 | 65043460 | 9.76G | 92.04 | 54.50 |
| M12F_3 | 62362836 | 60114474 | 9.02G | 92.20 | 55.16 |
