# Supplementary material for: Study on the transcriptome for breast muscle of chickens and the function of key gene RAC2 on fibroblasts proliferation
Source: BMC Genomics. 2021 Mar 6;22:157. doi: 10.1186/s12864-021-07453-0 (PMC7937270; doi:10.1186/s12864-021-07453-0)
Supplement: Supplementary file 8 — Additional file 8: Table S5. The results of comparison with reference genome for clean reads. [file 12864_2021_7453_MOESM8_ESM.docx]

Table S5 The results of comparison with reference genome for clean reads

| **Sample name** | **M4F_1** | **M4F_2** | **M4F_3** | **M8F_1** | **M8F_2** | **M8F_3** | **M12F_1** | **M12F_2** | **M12F_3** |
| --- | --- | --- | --- | --- | --- | --- | --- | --- | --- |
| Total reads | 57553102 | 60018588 | 59343986 | 50382466 | 54856958 | 59583598 | 55942770 | 65043460 | 60114474 |
| Total mapped | 43177424 (75.02%) | 44838256 (74.71%) | 43694835 (73.63%) | 38703348 (76.82%) | 41500712 (75.65%) | 45556986 (76.46%) | 46294890 (82.75%) | 53289902 (81.93%) | 48981380 (81.48%) |
| Multiple mapped | 2525503 (4.39%) | 2505471 (4.17%) | 2456730 (4.14%) | 2004292 (3.98%) | 2284665 (4.16%) | 2519220 (4.23%) | 2076345 (3.71%) | 2251607 (3.46%) | 2263595 (3.77%) |
| Uniquely mapped | 40651921 (70.63%) | 42332785 (70.53%) | 41238105 (69.49%) | 36699056 (72.84%) | 39216047 (71.49%) | 43037766 (72.23%) | 44218545 (79.04%) | 51038295 (78.47%) | 46717785 (77.71%) |
| Reads map to '+' | 20288169 (35.25%) | 21098271 (35.15%) | 20540828 (34.61%) | 18318135 (36.36%) | 19553810 (35.65%) | 21456278 (36.01%) | 22096601 (39.5%) | 25465835 (39.15%) | 23323113 (38.8%) |
| Reads map to '-' | 20363752 (35.38%) | 21234514 (35.38%) | 20697277 (34.88%) | 18380921 (36.48%) | 19662237 (35.84%) | 21581488 (36.22%) | 22121944 (39.54%) | 25572460 (39.32%) | 23394672 (38.92%) |
| Non-splice reads | 19256438 (33.46%) | 19882165 (33.13%) | 19048878 (32.1%) | 17186086 (34.11%) | 18313944 (33.38%) | 20868742 (35.02%) | 21510337 (38.45%) | 25965545 (39.92%) | 23770521 (39.54%) |

NOTES：(1) Total reads: The number reads of clean data; (2) Total mapped: The number reads located on the genome; (3) Multiple mapped: The number reads with multiple alignment sites on the reference genome; (4) Uniquely mapped: The number reads with unique alignment positions on the reference genome; (5) Reads map to '+'，Reads map to '-': The number reads compared to positive and negative chains in the genome; (6)Non-splice reads: The number reads aligned to one exon.
